# *Coronilla juncea*, a native candidate for phytostabilization of potentially toxic elements and restoration of Mediterranean soils

**DOI:** 10.1038/s41598-022-14139-4

**Published:** 2022-06-15

**Authors:** Alma Heckenroth, Pascale Prudent, Hélène Folzer, Jacques Rabier, Stéven Criquet, Arne Saatkamp, Marie-Dominique Salducci, Laurent Vassalo, Isabelle Laffont-Schwob

**Affiliations:** 1 Aix Marseille Univ, IRD, LPED, Marseille, France; 2 Fédération de Recherche ECCOREV N° 3098, Aix Marseille Univ, CNRS, Aix-en-Provence, France; 3 Aix Marseille Univ, Avignon Université, CNRS, IRD, IMBE, Marseille, France; 4 Aix Marseille Univ, CNRS, LCE, Marseille, France

**Keywords:** Ecophysiology, Restoration ecology, Environmental chemistry, Environmental impact

## Abstract

Soil contamination pattern due to industrial activities often leads to high concentrations of potentially toxic elements (PTE) decreasing with depth. This spatial heterogeneity of the soil contamination may have significant consequences on the soil properties and soil living communities. We evaluated the effects of both surface and solum soil contamination heterogeneity on *Coronilla juncea* L. (Fabaceae) functional traits in field conditions and the phytostabilization potential of this species. Plant and soil samples were collected on 3 sites along a PTE contamination gradient. The correlations between PTE concentration in plant and soil samples at 2 depths, physico-chemical properties of soil, plant biomass and soil microbial activity were tested. Field measurements highlight a decreasing PTE concentration with soil depth in addition to an important surface heterogeneity of As, Cu, Pb, Sb and Zn soil concentrations. Root PTE concentrations in *C. juncea* did not follow soil PTE concentrations. Concentrations of PTE in the root parts were higher than those of the aerial parts. Low PTE translocation and root symbioses with microorganisms suggest that this native plant species may play a role as engineer species with positive implications for the phytostabilization of Mediterranean PTE contaminated soils and their ecological restoration.

## Introduction

For the two last centuries, industrial activities have generated an accumulation of potentially toxic elements (PTE)^1^ in soils at the vicinity of activity areas^2^. Soil contamination by inorganic compounds coming from slap heaps and atmospheric deposits is a dissuasive limit for sustainable land uses in many parts of the world^3^. Indeed, PTE lead to ecotoxicological effects on plant and soil organisms and, microbial activities that can degrade soil functions, constrain vegetation recovery and be considered to constitute a human and environmental health risk. Phytoremediation techniques have been developed for many ecological contexts^4^. However, little is known on how spatial heterogeneity in contaminant distribution affects the efficiency of phytoremediation approaches. If industrial contaminations are often characterized by an important spatial heterogeneity^5–7^, previous research works mostly focused on topsoil (ca. 0–25 cm) contamination heterogeneity as a whole. The effects of contamination heterogeneity through the soil profile have been little investigated and approaches taking into consideration both spatial axes of contamination heterogeneity (surface and solum) are lacking. However, the heterogeneity of soil characteristics even at a small scale is known to influence the response of plants and their associated microorganisms in terms of structure and functions, from the processes linked to the root growth and nutrient resource capture to ecosystem functioning^8–11^. This soil heterogeneity contributes to the characteristic patchy distribution of vegetation in dry ecosystems, patches that in turn provide more favorable microenvironments for plant regeneration^12^. In industrial and urban contexts, spatial heterogeneity of the soil contamination is often considered as typical^7^. This is also the case for smelter activities that generate important PTE atmospheric emissions and then particular deposits at the topsoil surface of the surrounding environment^6,13,14^. The depth distribution of industrial contaminants throughout the soil profile shows often decreasing concentrations of PTE with depth, caused by atmospheric deposits and particle run-off^15,16^. These surface and solum heterogeneities of soil contamination may have significant consequences on soil functions and communities of living organisms. Recent works that studied the effects of contamination variability on composition and structure of plant communities found correlations between plant assemblages and PTE soil concentrations indicating an alteration of the ecosystem trajectory under pollution pressure^5,17–19^. Plants can deal with small-scale heterogeneity of contaminants by avoiding the most contaminated patches of soil^20^. This ‘rooting plasticity’ may have important consequences for efficiency of phytostabilization in sites where the contaminations are concentrated in topsoils.

Plant-soil-microorganism interactions in the rhizosphere are determinant for the ability of plant and associated microorganism communities to develop, by increasing the resistance of the host plant to biotic and abiotic stresses^21^. However, Giller et al.^22^ showed that microorganisms are more sensitive to stress caused by PTE contamination compared to other soil organisms. By affecting structure and functional diversity of microbial communities, PTE are likely to alter the biological processes of soils, such as litter decomposition, soil organic matter mineralization, and nutrient recycling^23,24^. Furthermore, inorganic contaminations affect interactions between plants and microorganisms in the rhizosphere^25^. Yet, these interactions are a major determinant of the plant tolerance mechanisms to contaminants, by influencing nutrient availability and PTE bioavailability and ecotoxicity^26^ and leading in some cases to a decrease of stress and toxicity generated by PTE^27^. Indeed, plant-associated microorganisms can contribute to a reduction of PTE in aboveground parts of plants by promoting the sequestration of contaminants in both roots and rhizosphere microorganisms^28^, immobilization in the soil^29^, or by direct root accumulation^30,31^ in continuous feedback linked to plant community composition and soil abiotic properties^32^. Phytostabilization approaches are based on the combined action of plants and their associated microorganisms for stabilization of inorganic contaminants and the physical protection against erosion provided by plant cover to reduce the transfer of these contaminants^33–36^. Thus, spatial heterogeneity of soil contaminants needs to be considered to avoid any failure in reducing PTE transfer. Mechanisms underlying the strategy of plant-microorganism to deal with contamination heterogeneity have to be elucidated. Moreover, the length of summer dryness of the soil in certain biogeographic areas such as the Mediterranean Basin added to steep slopes leading to soil erosion are local constraints increasing the risk of PTE transfer^37^. Besides, a recent research work on spatial distribution of PTE from smelters from Hunan Province of China contributed to a better understanding of PTE distribution in soil layers and demonstrated the effect of Zn, Cu, As on microorganism communities with the help of metagenomic methods^38^. Microorganisms are a key performance indicator of the soil quality and may provide information of soil degradation using methods from microbial biomass and activities to express soil functionalities and/or metagenomic to express soil biodiversity^38,39^. Consequently, to optimize phytostabilization of polluted soils, there is a need to take into consideration the spatial heterogeneity of the soil contamination and plant-microorganism response to PTE in soil^40,41^.

Phytostabilization approaches constitute a nature-based solution considered as adapted to the context of protected areas where no redevelopment of brownfields is considered. We selected a former lead smelter named Escalette located in the Calanques National Park (Marseille, south-east France). A previous study highlighted an important heterogeneity of the PTE topsoil contamination^19^ and, has led to a selection of native pseudo-metallophytes being potential candidates for phytostabilization. Pseudo-metallophytes refer to plant species having acquired PTE tolerance however being able to grow both in PTE polluted and non-polluted areas. They differ from metallophytes restricted to metal-rich soils^42^. Amongst those, the native leguminous *Coronilla juncea* L. appeared to tolerate high levels of PTE in soils. This species is known to form root symbioses with N-fixing bacteria favouring rehabilitation of arid degraded lands and considered as a nurse plant^43,44^. The current study focused on the phytostabilization potential of this plant species in the context of elevated PTE contamination heterogeneity in Escalette soils.

We further assume that the PTE soil concentration should be higher in the topsoil layer, as the industrial contamination of the area was mainly caused by atmospheric deposition of contaminants from the smelter activity, then by those contained in ashes from the degraded horizontal chimney located upstream of the contaminated studied area^38^. We were also expecting deleterious effects on microbial and plant traits depending on PTE soil concentrations^39^. We hypothesize deleterious effects may follow a hump-back model with intermediary PTE contamination being the less deleterious on biotic interactions (sensu Grime^45^).

Our purpose here was to investigate: (i) the effects of both surface and solum contaminant heterogeneities on root and microorganism functional traits; (ii) the phytostabilization efficiency under field conditions. We therefore conducted an analysis of PTE concentrations in plant and soil samples, the characterization of pedological properties and indirect measurements of soil microbial activity based on basal and substrate induced respirations (BR *vs* SIR) and estimation of the number of root nodules, depending on soil depth in a range of field conditions.

## Results

### Soil physico-chemical characteristics

**Figure 1:**
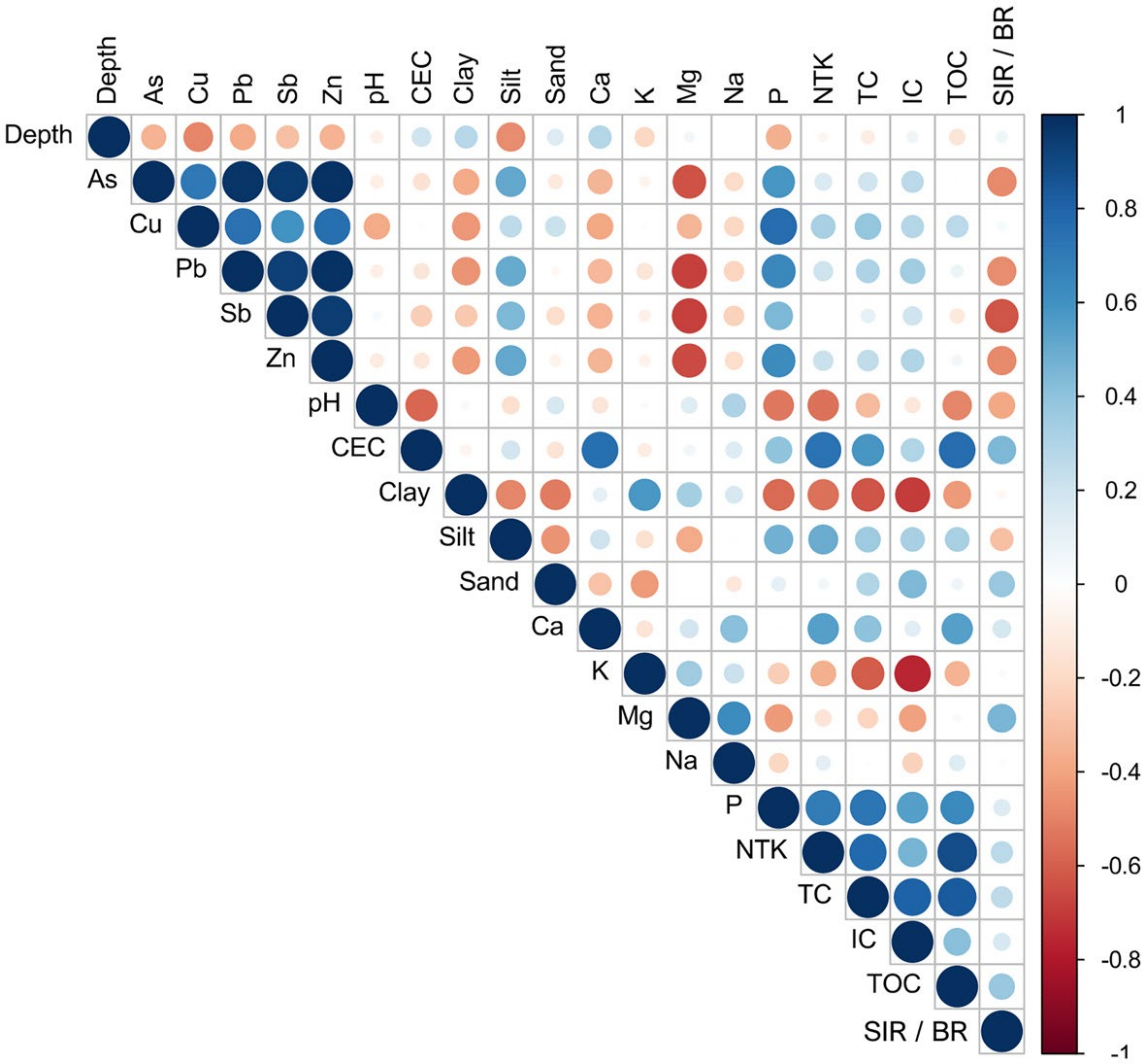
Correlogram of Spearman correlation test performed between pedological properties and soil PTE (metal and metalloid pseudo-total concentrations in mg/kg) measured at both soil depths (0–10 cm and 10–20 cm). Blue dots correspond to the positive correlations and red dots to the negative correlations. The size and color intensity of the dots are proportional to the correlation coefficient value. Parameters: CEC = cation exchange capacity; NTK = total Kjeldahl nitrogen; TC = total carbon; IC = inorganic carbon; TOC = total organic carbon; SIR/BR = substrate-induced respiration/soil basal respiration.

All soil samples showed similar pedological properties. Since no significant effect of soil depth was observed on the pedological properties, only the properties measured in the topsoil, between 0 and 10 cm depth, are given in Table 1. Cation exchange capacity (CEC) and MgO were significantly lower in Escalette 2 (E_2_) compared to Escalette 1 (E_1_) and Sormiou (S), respectively. Cation exchange capacity was positively correlated with total organic carbon (TOC; *r* = 0.8; p-value ≤ 0.001; Fig. 1). Apart the higher exchangeable Mg content, no significant difference of concentrations for nutritional elements and organic matter could be detected for soil from the control site to contaminated soils E_1_ and E_2_. The soil texture of all sites was mostly loam and silty loam (Table 1). Total organic carbon content ranged from 2.8 to 4.8%; however, no significant differences between sites could be revealed. Considering the results obtained for each site and at both soil depths (Fig. 1), TOC, total Kjeldahl nitrogen (NTK), cation exchange capacity and exchangeable P and Ca were altogether positively correlated (p-value ≤ 0.05; Fig. 1). The substrate-induced respiration/soil basal respiration (SIR/BR) ratio showed the lowest value at the most contaminated site E_2_, with an average increasing ratio around 490% vs 790% and 760% for S and E_1_, respectively (Table 1). Considering all sites and soil depths together, there were no significant correlations between BR, SIR neither SIR/BR and, other soil characteristics. But there were significant negative correlations between SIR/BR and As, Pb, Sb and Zn soil pseudo-total concentrations (p-value ≤ 0.05; Fig. 1).

**Table 1:** Soil pedological properties (mean ± standard deviation) of the studied sites measured between 0 and 10 cm depth and FAO soil classification.

Soil pedological properties	Sites		
	S	E_1_	E_2_
pH_water_	8.08 ± 0.03a	7.94 ± 0.10a	8.11 ± 0.12a
CEC (meq/100 g)	20.30 ± 3.00ab	24.82 ± 7.74a	16.20 ± 3.52b
Clay (%)	20.92 ± 6.26a	20.00 ± 6.02a	15.76 ± 5.52a
Fine silt (%)	19.08 ± 1.05a	16.3 ± 6.45a	19.36 ± 8.27a
Coarse silt (%)	27.16 ± 6.41a	35.78 ± 5.41a	33.06 ± 7.49a
Fine sand (%)	14.4 ± 3.89a	10.56 ± 3.12a	12.5 ± 2.04a
Coarse sand (%)	18.5 ± 3.79a	17.36 ± 5.75a	19.34 ± 6.06a
CaO (g/kg)	10.86 ± 1.48a	11.09 ± 1.62a	9.03 ± 1.31a
K_**2**_O (g/kg)	0.22 ± 0.05a	0.21 ± 0.055a	0.19 ± 0.05a
MgO (g/kg)	0.42 ± 0.10a	0.21 ± 0.06b	0.18 ± 0.06b
Na_**2**_O (mg/kg)	36.2 ± 14.52a	28.8 ± 10.38a	36.4 ± 25.70a
P_2_O_5_ (mg/kg)	26.6 ± 11.35a	59.8 ± 38.54a	37.8 ± 9.98a
NTK (mg/g)	2.85 ± 0.77a	3.88 ± 2.13a	2.69 ± 0.94a
TC (%)	7.12 ± 2.47a	9.28 ± 2.27a	6.85 ± 1.27a
IC (%)	3.23 ± 1.12a	4.51 ± 1.81a	4.04 ± 0.68a
TOC (%)	3.89 ± 1.48a	4.78 ± 2.66a	2.82 ± 1.04a
SIR/BR (%)	794 ± 51a	757 ± 121a	493 ± 136b
FAO soil classification	Calcisol	Calcisol	Calcisol

Means (n = 5) followed by different letters are significantly different (Wilcoxon test. p ≤ 0.05). Sites: S = reference site at Sormiou. E_1_ = Escalette 1 and E_2_ = Escalette 2. Parameters: CEC = cation exchange capacity; NTK = total Kjeldahl nitrogen; TC = total carbon; IC = inorganic carbon; TOC = total organic carbon; SIR/BR = substrate-induced respiration/soil basal respiration.

### Soil potentially toxic element contamination heterogeneity

Looking to the element concentrations within the topsoil between 0 and 10 cm depth, it was possible to distinguish two groups of elements: on one hand, As, Cu, Pb, Sb and Zn with concentrations that were significantly different among sites and following a decreasing trend i.e. E_2_ > E_1_ > S, out of which no significant difference of Cu concentrations were pointed out between E_1_ and E_2_ (Table 2); on the other hand, Al, Fe, Mn and Ti soil concentrations with no significant difference detected between the 3 sites. The analysis of the PTE concentrations globally showed an important heterogeneity of topsoils as indicated by high standard deviations (Table 2). As, Cu, Pb, Sb and Zn showed decreasing concentrations with soil depth (Table 3). Moreover, significant differences were observed for the variation of PTE concentrations between 0–10 cm and 10–20 cm depth when comparing the most contaminated site E_2_ and reference site S (Table 3). Concerning As and Zn, this difference between the two depths was also significant for all sites. Since this difference of concentration calculated between 0–10 cm and 10–20 cm soil depth quantifies the PTE contamination heterogeneity through the soil profile, it was quite clear that the most contaminated sites showed also the most heterogeneous vertical contamination regarding the data (Table 2). More globally, the contamination heterogeneity, both horizontal and vertical, seemed more marked on the site where PTE concentrations reached the highest levels. The phytoavailable PTE concentrations showed the same trend as pseudo-total ones with the highest values observed at the E_2_ site, then decreasingly E_1_ and S (only elements that showed different pseudo-total concentrations from one site to another are presented Table 3). At the reference site S, phytoavailable concentrations of As and Sb were below the detection limit. For Pb, a phytoavailable fraction of 4% was measured between 0 and 10 cm depth at this site. Cu phytoavailable concentrations were similar among sites, following the trend of the Cu pseudo-total concentrations. For the other PTE, the phytoavailable fractions seemed to vary depending on each element, site and depth: at Escalette sites (E_1_ and E_2_), phytoavailable fractions were between 3 and 9% for Sb, between 9 and 13% for As, 11% and 19% for Cu, between 17 and 21% for Zn, and from 25 to 49% for Pb. Cu phytoavailable fraction ranged from 14 to 19% at the reference site, and Zn fraction was 11% for both depths at the same site. The analysis of regressions between pseudo-total and phytoavailable concentrations showed significant positive correlations (p-value ≤ 0.001), such correlations being higher in the case of Sb and Zn (*r*^*2*^ > 0.9) compared to As and Pb (0.76 < *r*^*2*^ < 0.78). For Cu, the correlation coefficient *r*^*2*^ was around 0.6.

**Table 2:** Soil elements (PTE and other element pseudo-total concentrations in mg/kg) measured at 0–10 cm and 10–20 cm depth.

Soil PTE	Depth	Sites		
		S	E_1_	E_2_
Al	0–10 cm	31,926 ± 8261a	30,382 ± 10802a	34,621 ± 8900a
	10–20 cm	33,149 ± 8591a	27,945 ± 4622a	41,217 ± 10741a
As	0–10 cm	15.5 ± 3.5c	675 ± 262b	5945 ± 1974a
	10–20 cm	14.0 ± 1.9b	505 ± 207b	1745 ± 863a
Cu	0–10 cm	15.9 ± 2.3bc	23.7 ± 7.8b	30.5 ± 9.9ab
	10–20 cm	14.2 ± 1.5a	20.9 ± 7.5a	19.1 ± 10.3a
Fe	0–10 cm	23,590 ± 4885a	21,904 ± 5133a	23,989 ± 4737a
	10–20 cm	19,866 ± 2932a	21,400 ± 5003a	26,337 ± 6222a
Mn	0–10 cm	683 ± 130a	491 ± 252a	634 ± 149a
	10–20 cm	713 ± 143a	440 ± 185a	705 ± 156a
Pb	0–10 cm	90.9 ± 36.9c	6286 ± 2811b	42,320 ± 11251a
	10–20 cm	59.3 ± 17.9c	5624 ± 3775b	12,159 ± 5932a
Sb	0–10 cm	1.4 ± 1.31c	202 ± 38b	2589 ± 829a
	10–20 cm	1.1 ± 0.05c	191 ± 69b	885 ± 359a
Ti	0–10 cm	581 ± 79a	491 ± 88a	522 ± 30a
	10–20 cm	558 ± 118a	490 ± 74a	549 ± 67a
Zn	0–10 cm	105 ± 18.2c	1767 ± 760b	10,704 ± 4524a
	10–20 cm	96.1 ± 13.5c	1290 ± 720b	3898 ± 833a

In the same row, values (mean ± standard deviation (n = 5)) followed by different letters are significantly different (Wilcoxon test. *P* ≤ 0.05). Sites: S = reference site at Sormiou. E_1_ = Escalette 1 and E_2_ = Escalette 2.

**Table 3:** Variations (δ) of PTE (pseudo-total concentrations) between 0–10 cm and 10–20 cm depth within the same sample (δ = [PTE]_0–10_ – [PTE]_10–20_) and soil phytoavailable PTE concentrations (mean ± standard deviation (n = 5)).

Sites		Variations (δ) of PTE soil concentration between 0–10 cm and 10–20 cm				
		As	Cu	Pb	Sb	Zn
Sormiou (S)	Min	− 0.5	− 0.8	13	− 1.1	− 7.3
	Mean ± SD	5.5 ± 5.0 c	1.8 ± 2.5 bc	37 ± 23 bc	1.5 ± 1.7 bc	10.6 ± 20.9 c
	Max	11.5	4.8	66	2.6	40.0
Escalette 1 (E_1_)	Min	86	0.05	− 756	− 50	243
	Mean ± SD	151 ± 72 b	3.3 ± 2.6 b	732 ± 1113 b	10.5 ± 44 b	446 ± 223 b
	Max	230	6.6	1914	47	643
Escalette 2 (E_2_)	Min	3670	11.3	25,593	1281	4249
	Mean ± SD	4850 ± 1321 a	13.9 ± 4.1 a	34,116 ± 8069 a	1985 ± 614 a	8164 ± 3556 a
	Max	6441	20.0	44,991	2714	12,351

Sites	Depths	Phytoavailable PTE (mg kg^−1^) at 0–10 cm and 10–20 cm soil depth				
		As	Cu	Pb	Sb	Zn
Sormiou (S)	0–10 cm	< LOD	2.9 ± 1.3 (19%)	5.52 ± 7.01 (4%)	< LOD	11.13 ± 5.37 (11%)
	10–20 cm	< LOD	2.0 ± 0.8 (14%)	< LOD	< LOD	10.32 ± 3.4 (11%)
Escalette 1 (E_1_)	0–10 cm	78.3 ± 43.5 (11%)	4.1 ± 1.3 (17%)	3075 ± 1344 (49%)	7.88 ± 3.57 (4%)	325 ± 184 (17%)
	10–20 cm	49.7 ± 35.0 (9%)	3.0 ± 1.1 (14%)	2336 ± 1245 (45%)	4.86 ± 4.18 (3%)	241 ± 144 (19%)
Escalette 2 (E_2_)	0–10 cm	670 ± 376 (11%)	5.9 ± 2.2 (19%)	10,246 ± 2873 (25%)	234 ± 90 (9%)	2062 ± 918 (20%)
	10–20 cm	267 ± 266 (13%)	2.4 ± 2.8 (11%)	4818 ± 3163 (41%)	74 ± 35 (8%)	750 ± 269 (21%)

For phytoavailable PTE, the percentage in brackets refers to the ratio of the phytoavailable PTE concentrations on pseudo-total PTE concentrations ([PTE]_phytoavailable_/[PTE]_pseudo-total_). “ < LOD” indicates values below detection limits. In the same row, values followed by different letters are significantly different (Wilcoxon test, *P* ≤ 0.05).

### Accumulation and transfer of PTE in plants

There were no significant differences between PTE concentrations in root parts of *C. juncea* from different soil depths for both contaminated sites E_1_ and E_2_ (Table 4), in contrast to the PTE concentrations measured in the soils. There were generally higher concentrations of PTE in the root parts compared to the aerial parts, especially at the most contaminated site E_2_ and with the major concentration differences observed between aerial parts and root parts from the topsoil layer (0 to 10 cm). Furthermore, the highest plant concentration values of As, Pb, Sb and Zn were always measured in the root parts located within the topsoil, where the PTE contaminations originated from past industries were also the highest. Nevertheless, no significant differences between PTE mean concentrations of the roots sampled at different depths were detected. At the reference site S, significant differences were only measured for Cu, Fe, Mn and Ti between root parts from both depths with higher concentrations in the deepest samples (from 10 to 20 cm below the surface). Bioconcentration factors (BCF) showed increasing values with increasing soil depth (Table 5), highlighted by a positive correlation: this relation was significant considering the BCF of As, Pb, Sb and Zn, for which Spearman’s *r* was respectively 0.55, 0.55, 0.66 and 0.63 (p-value < 0.05). The BCF were also negatively correlated to the PTE pseudo-total soil concentrations with *r* equal to − 0.69, − 0.66, − 0.64, − 0.53 and − 0.62 for As, Cu, Pb, Sb and Zn respectively (p-value < 0.05). There were no significant correlations between the root concentrations and BCF for the industrial-originated elements As, Pb, Sb and Zn but a positive one for Al, Cu, Fe, Mn and Ti (0.56 ≤ *r* ≤ 0.93; p-value < 0.05). Amongst all the BCF, Cu was the element for which the maximum values were measured.

**Table 4:** PTE and other element concentrations measured in the aerial parts (AP) ant root parts (RP) of the individuals of *Coronilla juncea* at the different sites (S, E_1_, E_2_) and soil depths.

Site, organ and depth	PTE and other elements concentrations (mg kg^−1^)								
	Al	As	Cu	Fe	Mn	Pb	Sb	Ti	Zn
**Sormiou (S)**									
S AP	326.9 ± 223.1 a	< LOD	9 ± 3.5 a	146.3 ± 65.4 bc	31.4 ± 10.2 a	3.5 ± 4.1 a	< LOD	3.2 ± 1.5 b	70.8 ± 36.6 a
S RP 0–10 cm	345.3 ± 265 a	< LOD	0.8 ± 0.7 b	35 ± 78.3 c	< LOD	1.2 ± 1.2 a	< LOD	4.4 ± 1.9 b	15.3 ± 14.7 b
S RP 10–20 cm	631.8 ± 372.9 a	< LOD	7.2 ± 4.1 a	444.5 ± 269.4 a	17.6 ± 6.4 b	2.4 ± 1.7 a	< LOD	8.8 ± 5.3 a	17.8 ± 15.2 b
*Average RP*	*377.1* ± *331.7*	< *LOD*	*2.3* ± *4.4*	*129.2* ± *277.2*	*5.0* ± *9.6*	*1.1* ± *1.4*	< *LOD*	*2.8* ± *5.4*	*14.0* ± *14.1*
**Escalette 1 (E**_**1**_**)**									
E_1_ AP	162.4 ± 79.6 b	4.9 ± 4.1 a	4.2 ± 0.7 a	117.9 ± 51.3 b	26.0 ± 3.7 a	59.7 ± 29.9 a	2.3 ± 1.6 a	2.3 ± 1.3 b	88.6 ± 28.9 a
E_1_ RP 0–10 cm	739.8 ± 569.4 a	22.8 ± 17.5 a	7.8 ± 4.3 a	482.6 ± 343.1 a	15.8 ± 10.3 a	271.6 ± 183.4 a	6.3 ± 3.7 a	9.9 ± 8.6 a	97.4 ± 62.3 a
E_1_ RP 10–20 cm	1736.6 ± 32.5 ab	50.5 ± 19.6 a	5.7 ± 1.2 a	1116.9 ± 74.4 ab	22.9 ± 1.9 a	463 ± 251.7 a	13 ± 3.1 a	22.7 ± 1.3 ab	127.1 ± 47.6 a
*Average RP*	*693.6* ± *673.0*	*22.6* ± *21.2*	*6.57* ± *3.6*	*462.0* ± *418.5*	*13.9* ± *9.1*	*271.7* ± *204.2*	*6.1* ± *2.6*	*8.8* ± *9.4*	*93.3* ± *56.3*
**Escalette 2 (E**_**2**_**)**									
E_2_ AP	140.1 ± 119.8 b	9.4 ± 11.8 b	3.8 ± 0.9 b	90.9 ± 78.4 b	30.2 ± 7.9 a	86.5 ± 91.1 b	5.4 ± 4.3 b	1.8 ± 1.7 b	138.7 ± 18.7 a
E_2_ RP 0–10 cm	718.2 ± 341.5 a	96.4 ± 50.4 a	6.6 ± 2.6 a	498.7 ± 241.8 a	16.9 ± 7.3 a	742.4 ± 405.1 a	44.5 ± 23.1 a	10.5 ± 4.7 a	280.7 ± 161.3 a
E_2_ RP 10–20 cm	1706.6 ± 1105.5 ab	38.3 ± 2.4 ab	11 ± 7.6 ab	1104.3 ± 744.4 ab	30.5 ± 7.8 a	310.2 ± 37.8 ab	35.1 ± 3.4 ab	25.4 ± 14.3 ab	208.7 ± 66.3 a
*Average RP*	*677.3* ± *717.0*	*71.7* ± *49.9*	*6.0* ± *4.3*	*463.2* ± *467.6*	*16.3* ± *9.5*	*553.3* ± *392.6*	*25.8* ± *24.6*	*10.1* ± *10.0*	*233.1* ± *138.9*

The average root part concentrations (in italics) have been calculated using mean concentrations and root biomasses per depth. For a same element and a same site, different letters indicate a significant difference between sites, organs and/or depths (Wilcoxon test, p-value ≤ 0.05).

**Table 5:** Bioconcentration factors (BCF) i.e. ratios of *Coronilla juncea* root PTE concentrations vs. soil PTE pseudo-total concentrations for As, Cu, Pb, Sb and Zn.

	Site	S		E_1_		E_2_	
	Depth	0–10 cm	10–20 cm	0–10 cm	10–20 cm	0–10 cm	10–20 cm
BCF As (%)	Min		0.94	5.66		0.69	2.56
	Mean ± SD	< LOD	3.23 ± 2.10	7.79 ± 3.02	< LOD	1.69 ± 1.14	4.07 ± 2.14
	Max		6.06	9.93		3.59	5.58
BCF Cu (%)	Min	< LOD	18.25	25.68	29.73	11.89	39.04
	Mean ± SD	3.77 ± 8.44	34.06 ± 19.25	25.85 ± 0.24	52.16 ± 35.04	23.64 ± 13.31	92.06 ± 74.98
	Max	18.87	67.18	26.02	104.04	45.10	145.07
BCF Pb (%)	Min	< LOD	1.71	5.84	0.00	0.74	3.05
	Mean ± SD	1.42 ± 1.56	4.16 ± 2.43	8.04 ± 3.11	4.68 ± 3.80	1.76 ± 1.10	4.56 ± 2.15
	Max	3.78	7.52	10.23	8.12	3.59	6.08
BCF Sb (%)	Min		0.98	4.99		0.80	4.11
	Mean ± SD	< LOD	3.18 ± 1.83	6.82 ± 2.58	< LOD	1.74 ± 1.05	6.46 ± 3.32
	Max		5.60	8.64		3.52	8.80
BCF Zn (%)	Min	< LOD	2.44	5.96	8.23	1.39	6.31
	Mean ± SD	12.43 ± 13.60	5.25 ± 2.60	7.87 ± 2.71	19.66 ± 18.53	2.75 ± 1.93	9.03 ± 3.85
	Max	35.22	8.95	9.79	47.35	6.14	11.76

Min: minimum; Max: maximum; Means (n = 5); SD: standard deviation. Sites: S = reference site at Sormiou. E_1_ = Escalette 1 and E_2_ = Escalette 2.

Translocation factors (TF) for the industry-related PTE were the highest for As, Pb, Sb and Zn at the E_2_ site (Table 6). At the most contaminated site E_2_, maximum TF values for As, Pb and Sb ranged from 25.60 to 28.95% and seemed lower than maximum TF values of Cu and Zn at 86.76 and 186.05%, respectively. For all data measured at Escalette sites, root concentrations for As, Cu and Pb were negatively correlated to Zn translocation factor (− 0.73 ≤ *r*  ≤ − 0.68; p-value < 0.05). For all correlations calculated between the TF and PTE root concentrations (within the topsoil), *r* coefficients were systematically negative but not significant, except for the ones mentioned above and, also between TF for Cu and Cu and Zn concentrations in the root parts (*r* = − 0.88 and *r* = − 0.69 respectively; p-value < 0.05).

**Table 6:** Translocation factors (TF) in *Coronilla juncea* individuals i.e. ratios of PTE aerial part concentration vs. PTE root concentration in the topsoil (0 to 10 cm depth) for As, Cu, Pb, Sb and Zn.

		Sites		
		S	E_1_	E_2_
TF As (%)	Min		5.83	1.61
	Mean ± SD	< LOD	67.00 ± 123.45	9.49 ± 9.82
	Max		287.75	25.60
TF Cu (%)	Min	0.00	35.58	39.65
	Mean ± SD	20.46 ± 68.12	65.59 ± 29.49	61.73 ± 19.71
	Max	152.32	98.81	86.76
TF Pb (%)	Min	0.00	11.06	3.58
	Mean ± SD	55.75 ± 57.03	56.62 ± 91.14	12.33 ± 10.34
	Max	127.06	219.55	28.95
TF Sb (%)	Min		14.80	4.01
	Mean ± SD	< LOD	48.19 ± 52.62	13.32 ± 8.66
	Max		141.31	26.84
TF Zn (%)	Min	0.00	42.77	22.80
	Mean ± SD	402.92 ± 321.34	186.65 ± 198.34	75.76 ± 64.56
	Max	685.81	506.08	186.05

Min: minimum; Max: maximum; Means (n = 5); SD: standard deviation. Sites: S = reference site at Sormiou. E_1_ = Escalette 1 and E_2_ = Escalette 2.

### Effect of the soil contamination heterogeneity on plant development and its associated microorganisms

**Figure 2:**
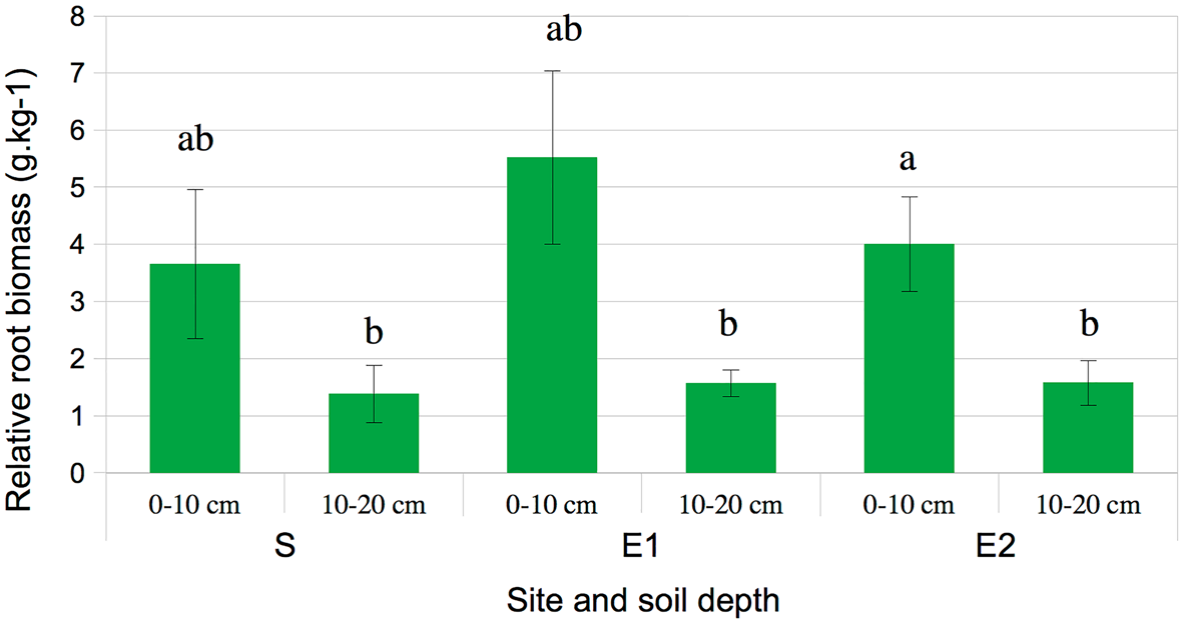
Relative root biomass (mean ± standard deviation) i.e. ratio of the dry root biomass of *Coronilla juncea* vs. dry soil mass at different soil depths and sites. Means are expressed in gram of dry roots per kg of dry soil. Different letters indicate a significant difference between means (p-value ≤ 0.05). Sites: S = reference site at Sormiou. E_1_ = Escalette 1 and E_2_ = Escalette 2.

**Figure 3:**
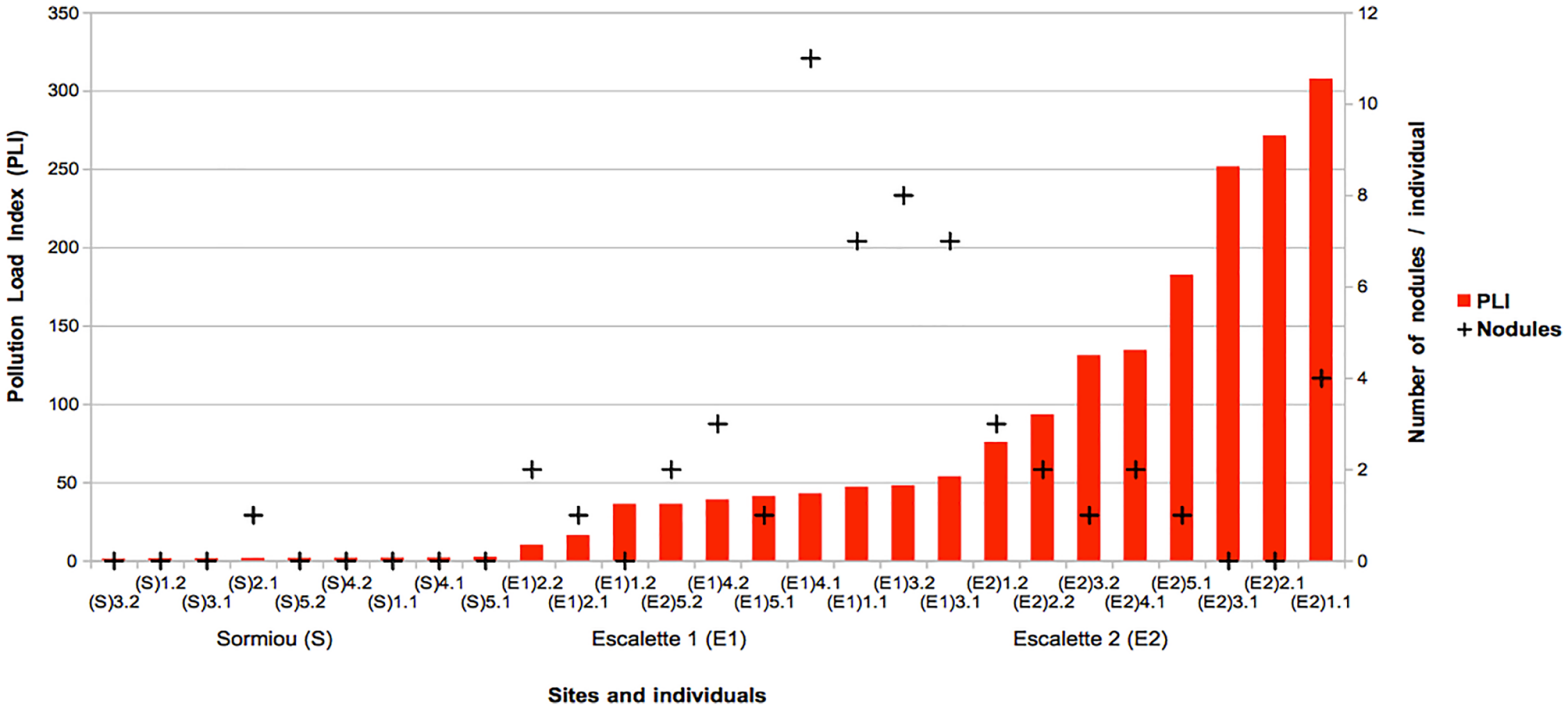
Number of nodules (black crosses) in root samples at different soil depths in regard to an increasing pollution load index (red bars) gradient in the soil. On the axis X, the sample code corresponds to the site (first letter), the individual (first number) and, the depth (second number): i.e. S3.2 = Sormiou. Individual 3. Depth: 10–20 cm.

Even if the mean values of root biomass seemed higher in all topsoils compared to deeper soils, it was only significantly different at E_2_ site (Fig. 2). Considering all sites and depths, positive correlations were observed between PTE soil concentrations and root biomasses (0.44 ≤ *r* ≤ 0.58; p-value < 0.05) with no clear evidence of an inhibition of the root growth by contaminants. Because of the small sample size and an important variability (i.e. only one nodule observed in all Sormiou root samples, from 0 to 11 nodules per root samples in E_1_ site, and from 0 to 4 nodules, in E_2_ site), no significant differences were detected on nodules occurrence between the different conditions. Nevertheless, it was obvious that number of root nodule was more important in the root samples from Escalette sites than S site: from the 5 individuals of *C. juncea* at each Escalette site we collected a total of 40 nodules at E_1_ site and 15 nodules at E_2_ vs. 1 nodule at Sormiou site (S). Spearman correlation tests performed from all sites data confirmed these significant relations as nodule occurrence was positively correlated with pseudo-total soil concentrations of As, Pb, Sb and Zn (*r* = 0.51; 0.53; 0.46 et 0.53 respectively; p-value ≤ 0.05). Similar results were obtained for phytoavailable PTE soil concentrations (results not showed; p-value ≤ 0.05). However, at both Escalette sites, there were negative correlations observed between the number of nodules and the mobile fraction of As, Cu and Sb (− 0.65 ≤ *r* ≤ − 0.52; p-value < 0.05), also with pH (*r* = − 0.53; p-value < 0.05). Number of root nodules was positively correlated with soil carbon concentration, mostly total C (*r* = 0.77; p-value < 0.001) and total organic C (*r* = 0.76; p-value < 0.001), and NTK (*r* = 0.73; p-value < 0.001). Furthermore, the distribution of nodules in regard to increasing PTE soil concentrations (expressed as pollution load index—PLI) seemed to follow a trend (Fig. 3): there were more nodules per root sample at both depths at medium level of TPE contaminations (corresponding mostly to Escalette 1 (E_1_) samples). We observed an absence of nodules in roots both for most of the non-contaminated samples from Sormiou (S) and for some of the most contaminated points at Escalette 2 (E_2_), that could reveal plant tolerance mechanism to face PTE contamination linked to N-fixing bacteria.

## Discussion

Our field results clearly highlighted the spatial distribution of industry-related PTE depending on soil location and depth. A surface heterogeneity of As, Cu, Pb, Sb and Zn soil concentrations was observed. A decreasing trend in surface contamination was reported when increasing the distance to the source i.e. the brownfield of Escalette. Highly contaminated sites also showed a heterogeneous solum PTE contamination with higher levels of industry-related PTE in topsoils. Such a solum PTE distribution trend was previously demonstrated at a local scale by Heckenroth et al.^19^ and clearly correlated to the distance to the ruins of the smelter chimney. Our results are also in agreement with those mapped at the scale of the massif of Marseilleveyre^46^ demonstrating the effect of wind and uneven relief on PTE dispersion. Our results sustain the idea of a highest contamination of the topsoil layer, which was also observed by Cabrera et al.^15^ in Spain, Imperato et al.^16^ in Italy and Li et al.^38^ in China considering urban and industrial contaminations. Moreover, our results are in agreement with those also observed in the massif of Marseilleveyre by Gelly et al.^47^ in which isotopic analyses were conducted and confirmed that Pb and Zn in soils profiles up to 7 km from the brownfield were directly linked to the industrial activity whereas Cu contamination of the soils could not be linked to this activity.

Moreover, physico-chemical analyses of the natural geochemical environment conducted at both reference and contaminated sites and through the solum revealed quite constant values typical to local Mediterranean alkaline coastal soils^48,49^. It highlighted a relative similarity concerning the natural composition of the soils at the scale of the study area, underpinning the choice of the sites for comparing pollution levels. Furthermore, the properties that appeared to be the most spatially heterogeneous considering both surface soils and solum were those linked to the industrial contamination, represented by exogenous trace metal and metalloids. Some parameters linked to physico-chemical and biological properties of the soil showed some differences between sites, but globally, physico-chemical soil characteristics seemed to have a negligible influence on PTE mobility and, PTE concentration levels showed little impact on soil physico-chemical characteristics. Those characteristics were representative of calcareous Mediterranean soils and of results previously obtained in the same biogeographical context^14,50^ i.e. Mediterranean oligotrophic soils, with low content of nutrients and organic matter, and high level of carbonates, the latter considered as reducing PTE mobility^51^ and host a plant cover adapted to this harsh habitat with majorly stress-tolerant plant species^52,53^.

Our study aimed at identifying deleterious effects of PTE soil concentrations on microbial and *C. juncea* traits. A significant negative correlation between Sb and respiration (SIR/BR ratio) was measured in agreement with Sb toxicity effects on local soil microbial communities observed in a previous study in the massif of Marseilleveyre^54^ corroborating the toxic effect of this PTE recently examined on Chinese soils spiked with Sb at two different speciations (Sb(III) and Sb(V))^55^. Moreover, Guillamot et al.^54^ also highlighted that the Sb toxicity effect on microbial community was more important on non-contaminated soils involving inherited tolerance mechanisms for the local soil microbial communities that develop on contaminated sites. It seems consistent with the results obtained here on SIR/BR: the absence of significant difference between the reference site S and contaminated site E_1_ could be related to the tolerance of microbial communities; as SIR/BR appeared to be significantly lower at E_2_ site, which could indicate toxicity effects due to higher PTE contamination.

However, the number of nodules in the *C. juncea* root systems was not reduced in the most contaminated site and no significant reduction of *C. juncea* growth has been revealed depending on PTE. These results may confirm the PTE tolerance of this pseudo-metallophyte.

The tolerance of *C. juncea* to PTE might be linked to its root symbiosis with nitrogen-fixing bacteria as previously reported for other leguminous^56,57^. Our data sustain this idea by the higher number of nodules in root parts of plants located in the contaminated area. More precisely, we observed an absence of nodules in roots for most of the samples from the non-contaminated site Sormiou (S) and a small number of nodules for the most contaminated points at Escalette 2 (E_2_). The presence of nodules can be correlated to the soil P availability as it can enhance nodule development in N-fixing legumes^58^. P was positively correlated to PTE soil concentrations in this study, these results could also reveal plant and microorganism tolerance mechanisms to PTE contamination linked to N-fixing bacteria, as well as ecotoxicological effects linked to increasing PTE concentrations^59,60^.

Our results demonstrated that root PTE concentrations in *C. juncea* did not follow the solum PTE concentrations since no significant differences of PTE accumulation in roots were observed between the different depths from the same site (E_1_ or E_2_) despite a gradient of soil contamination. Moreover, concentrations of PTE in the root parts were higher than those of the aerial parts. *C. juncea,* by reducing PTE transfer into the food web, may also assist the reestablishment of the ecosystem^61^. These traits are characteristic of good candidates for phytostabilization with a plant tolerance to a wide range of soil PTE concentrations. Furthermore, *C. juncea* may act as an ecosystem engineer^62^ by increasing soil nitrogen content via rhizobial nitrogen fixation^63^. Consequently, it may function as a nurse species and favour the establishment of other plants species. Padilla et al.^64^ previously showed the usefulness of *C. juncea* for restoration in arid environments.

Using native plants for in situ phytostabilization purposes implies to get a strong knowledge about plant strategies in the field, which can be assessed by measuring root traits like PTE uptake, biomass and properties linked to symbiotic microorganisms^65^. These parameters may vary in relation to spatial heterogeneity contamination, which can be both of surface and solum. Under field conditions, response of plants to this spatial heterogeneity will also determinate their suitability and efficiency for PTE stabilization.

Many phytoremediation projects focus on response of plants in pot experiments using homogeneous soil samples under controlled conditions^42,66,67^. However, they seem little representative of the high heterogeneity under field conditions in contaminated areas or the complexity of biotic interactions. In the context of the highly constrained field conditions of our study area, i.e. shallow soil characterized as “skeletal”, high contamination by PTE and semi-arid climatic conditions^37^, it is necessary to go further in the understanding of microsite conditions and related responses of the plant and their associated microorganisms at different soil depths to select an adapted phytostabilization strategy. Furthermore, it is a known response of individual plants to use avoidance strategies in relation to heterogeneous soil contamination: for instance, in the case of atmospherically deposited PTE contaminants, topsoil layer where the pollutants are the most concentrated may be avoided by roots^20^, and consequently phytostabilization will not be optimal.

The sheer presence of *C. juncea* on the extremely contaminated Escalette site already demonstrated its tolerance to PTE contamination in a previous study on plant communities^19^. The results of this study highlighted the ability of *C. juncea* to tolerate contaminants and to grow in a wide range of soil PTE concentrations. PTE contamination exerted neither significant nor visible negative effects on plant biomass, even under increasing uptake of PTE by the plant. Bioconcentration factors (BCF) in aerial parts were always < 1 for industrial PTE, revealing restricted transfer of PTE from soil to roots, depending on soil PTE concentration level. The lowest translocation factors (TF), i.e. transfer of PTE from roots to shoots, were measured at the most contaminated site E_2_. Thus, the translocation restriction seemed to evolve depending on the concentration of PTE contained in the roots.

Many studies on contaminated soils only consider the effects of single stressors on isolated endpoints^68^. Our study integrated a pool of PTE, some of them originating from industrial activities, and considered two biological components i.e. plants and soil microorganisms. *C. juncea* tolerance to PTE was confirmed, as well as the soil resilience ability in the context of both Escalette sites, even if some toxic effects seemed to limit microbial activity at the most contaminated site E_2_.

## Conclusions

In a context of high surface and solum heterogeneity of soil contaminants from past industrial activities, *Coronilla juncea* seems a good candidate for phytostabilization in its Mediterranean distribution area. Low translocation of contaminants and root symbiosis with nitrogen fixing microorganisms suggest that *Coronilla juncea* may play a prime role for the restoration of contaminated ecosystems at Escalette and other Mediterranean brownfields by promoting soil quality.

## Methods

All the experiments comply with relevant institutional, national, and international guidelines and legislation.

### Study sites and sampling

**Figure 4:**
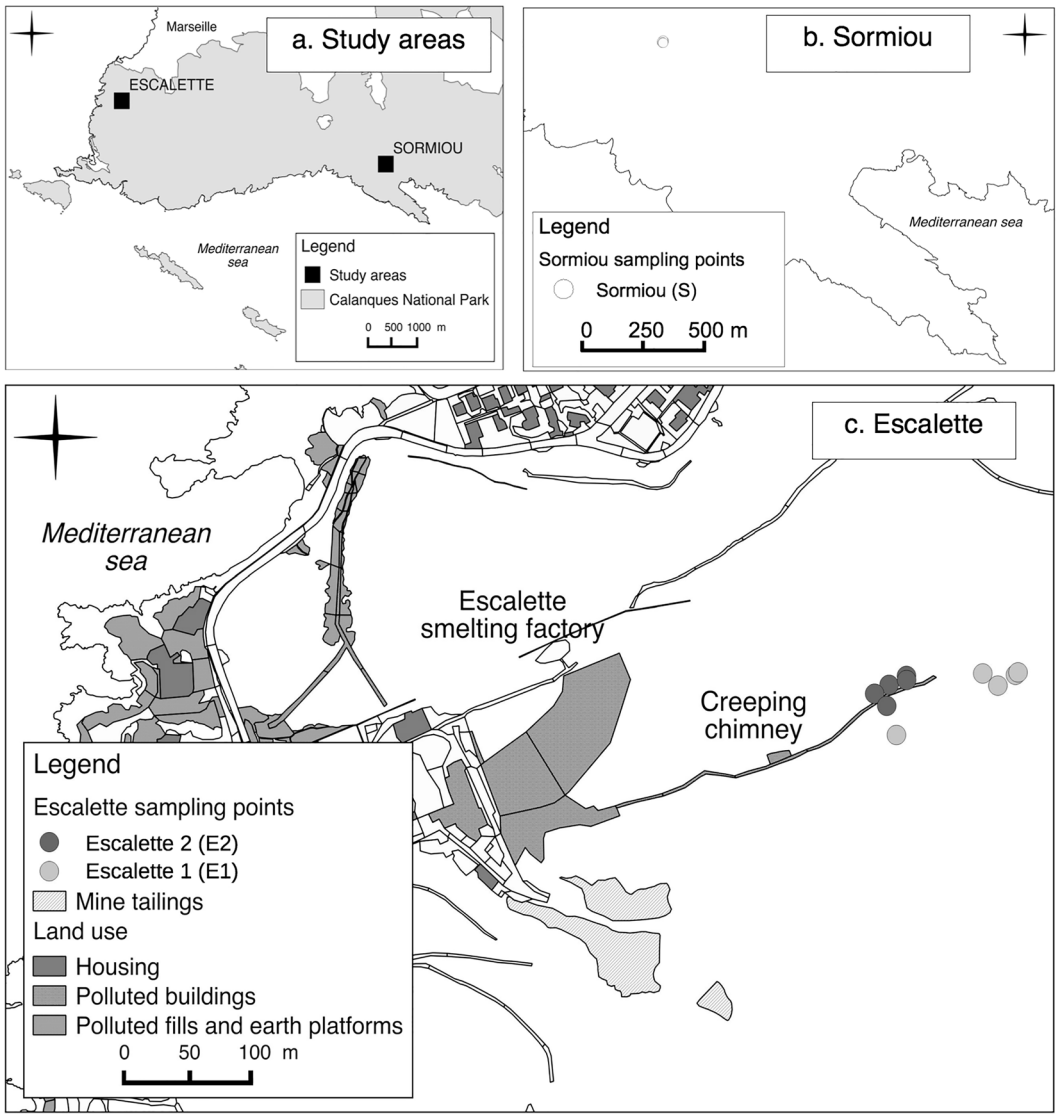
Location of the Escalette and Sormiou study areas (**a**); location of the sampling points S at the reference site Sormiou (**b**) and location of the sampling points E_1_ and E_2_ at the Escalette contaminated site (**c**). Calanques NP = Calanques National Park area. Map created using QGIS including data collected by PJ Dumas and A Heckenroth.

We studied two sites in the Calanques National Park (43.21357° N, 5.36735° E) at Sormiou and Escalette (Fig. 4), composed by a mosaic of spontaneous plant communities that correspond to calcareous xero-thermophilous shrublands (Mediterranean matorral), grasslands and scarce stands of *Pinus halepensis* Mill. Both sites have a semi-arid, Thermo-Mediterranean climate with average annual rainfall of 500 mm and average annual temperature of 16 °C^69,70^. Sites are at 120 to 140 m altitude. Escalette site is a brownfield of a former lead (Pb) smelter. PTE transfers, mostly composed of Pb, zinc (Zn), arsenic (As), copper (Cu) and antimony (Sb), are generated by erosion of the contaminated soils and degraded smelter buildings, under the influence of local strong winds and water runoff. Cadmium (Cd) has not been identified as a PTE in this study due to its soil content below level of detection in all previous studies. Previous studies at Escalette site and surroundings showed a high heterogeneity of surface soil contamination and high PTE tolerance of the native vegetation^14,19,71–75^.

Using previous measurements of PTE topsoil concentrations and phyto-ecological inventories on the field^14^, we preselected measure points where *C. juncea* individuals were abundant. We conducted measurements of the topsoil PTE contamination at the foot of the plants using a portable X-ray fluorescence analyzer (Niton^®^, xl3t GOLDD; Thermo Scientific). In order to avoid extreme values, only the points corresponding to Pb concentrations between the first and third quartiles have been conserved. We discarded plants growing directly in calcareous rocks. Sampling points were split into two groups at the Escalette site (Fig. 4a,c): “Escalette 1” (E_1_) corresponding to the lowest PTE concentrations and “Escalette 2” (E_2_) corresponding to the highest PTE concentration range located downstream of the degraded horizontal chimney. We used a third site at Sormiou (S) as the reference (Fig. 4a,b) representative of the local PTE soil background concentrations in the Calanques National Park. All sampling (soil and plants) were done according to the authorization given par the Calanques national park which restricts scientific sampling on its protected area.

For each study site, five replicates corresponding to a whole plant-soil sampling were selected. Aerial parts of *C. juncea* were cut at the collar and collected. We then sampled roots and soil at a 15 cm radius around the collar: two samples of roots and soil, from 0 to 10 cm depth, and from 10 to 20 cm depth. We kept all plant and soil samples at 4 °C in the lab until analysis. Soil samples were sieved through a 2 mm sieve and root parts were isolated from the soil with laboratory forceps.

### Soil analysis

#### Soil physico-chemical characteristics

Soil samples were dried at room temperature for the analysis of physico-chemical properties and PTE analyses. pH (ISO 10390), total Kjeldahl nitrogen (NTK, ISO 11261) and total organic carbon contents (TOC, ISO 10694) were analyzed by Laboratoire Développement Méditerranée (COFRAC accreditation n° 1-5865). Soil texture was determined using five fractions without decarbonatation (NF X31-107): clay (< 2 µm), fine silt (2 µm to 20 µm), coarse silt (20 µm to 50 µm), fine sand (50 µm to 0.2 mm) and coarse sand (0.2 mm to 2 mm). Available phosphorus (ISO 11263), exchangeable cations (CaO, K_2_O, MgO, Na_2_O, ISO 23470) and cation exchange capacity (CEC, ISO 23470) were also determined by Laboratoire Développement Méditerranée (COFRAC accreditation n° 1-5865). An aliquot of each soil sample was kept for PTE analyses as pseudo-total and EDTA extractable fraction.

#### Soil potentially toxic element analysis

Soil dried samples were ground by a RETSCH zm 1000 grinder with tungsten blades and titanium sieve to 0.2 mm particles, before analyses of the PTE concentrations. To obtain pseudo-total PTE extracts, we mineralized soils in three replicates in a microwave mineralizer (Milestone Start D) using aqua regia (1/3 HNO_3_ + 2/3 HCl). A ratio of dry soil/aqua regia solution corresponding to 1/20 w/v was used. Furthermore, to obtain an assessment of phytoavailable PTE, we used a solution of EDTA (0.05 mol L^−1^) with pH adjusted to 7 at a ratio of dry soil/EDTA corresponding to 1/10 w/v^76^. The solution was agitated at room temperature in an orbital shaker at 125 rpm for 1 h (Fisher Bioblock Scientific SM30B). Then, the EDTA extracts were centrifuged for 10 min at 8000 rpm (JP Selecta Medifriger BL-S). All the mineralization and extract products were filtered with a 0.45 μm-mesh, and the PTE concentrations were determined by inductively coupled plasma-atomic emission spectroscopy (ICP-AES, Jobin Yvon Horiba Spectra 2000). Quality assurance–quality controls and accuracy were checked using standard soil reference materials (CRM049–050, from RTC, USA) with accuracies within 100 ± 10%. We calculated contamination factors from PTE concentrations measured in E_1_ and E_2_ compared to the background PTE. Then, these contamination factors were used to estimate a multi-contamination level expressed as a pollution load index (PLI) allowing to estimate a multi-contamination level^34,69,72^.

#### Soil microbial activity

We estimated soil microbial activity by measuring the soil basal respiration (BR), i.e. CO_2_ released, using 10 g of sub-samples at 60% of their water holding capacity (WHC) weighed in 117 mL glass flasks (presumed saturating quantity). After internal atmosphere replacement, flasks were closed hermetically and incubated for 60 min at 20 °C. The CO_2_ released in the flasks was measured by gas chromatography (GC, Chrompack CHROM 3-CP 9001) and with a PorapakTM column containing 650 mL h^−1^ helium circulating. The substrate-induced respiration (SIR), i.e. CO_2_ released when substrate is added (g CO_2_ h^−1^ g^−1^ DW), was measured according to Anderson and Domsch^77^, using same method than for BR and by adding 50 mg of powdered glucose to each soil sample. Samples were pre-incubated for 90 min to reach a maximum rate of SIR. Then, after internal atmosphere replacement, flasks were closed hermetically and incubated for 60 min at 20 °C. The CO_2_ released in the flasks was measured in the same way than for BR. The growth of microbial activity was estimated by calculating the ratio between basal and substrate-induced respiration SIR/BR.

### Plant and root nodule analyses

Roots were separated from soil samples, washed under tap water to remove soil particles, then carefully rinsed with deionized water and dried using paper towel. For each root sample, the total number of nodules has been reported. Root and aerial parts of *C. juncea* were weighted within few hours of the return from the field to obtain the fresh biomass. The plant samples were dried at 60 °C for at least 72 h and weighed again to obtain the dry biomass. To analyze the plant PTE concentration, the dry samples were ground at 0.5 mm: the aerial parts using tungsten carbide blades (Foss Tecator Cyclotec 1093) and the root parts using a mixer mill (Retsch MM400). About 0.5 g dry matter of each plant sample (triplicates) was digested in microwave mineralizer system (Milestone Start D) with an acidic mixture (2/3 HNO_3_ + 1/3 HCl). After a filtration using a 0.45 µm mesh, the solutions were analyzed by ICP-AES as previously described for soil samples. Standard plant reference material (DC 73349 from NCS, China) was analyzed as a part of the quality assurance-quality control protocol (accuracies within 100 ± 10%).

### Data processing and statistical analyzes

In order to estimate the variation of soil PTE pseudo-total concentrations, we calculated differences of concentration (delta values) between samples of 0–10 cm and 10–20 cm depth within a plot (δ = [PTE]_0–10_ − [PTE]_10–20_). For comparing among sites, we chose to use delta values rather than directly soil pseudo-total concentrations per depth, that showed a high intra-site variability.

Root bioconcentration factors (BCF) were calculated from the soil and root PTE pseudo-total concentrations as following^67^: BCF_root_ = [PTE]_root parts_/[PTE]_soil_ where [PTE] is the PTE pseudo-total concentration. The translocation factor (TF) was estimated as following: TF = [PTE]_aerial parts_/[PTE]_root part_.

We tested for normality using a Shapiro–Wilk test in R 3.0.2. software^78^ and stats and ade4 packages^79^. When data were not normally distributed, we used Wilcoxon tests to analyze the differences between samples from different sites and/or depths (soil and root samples). We also used Spearman rank correlation tests to analyze the correlations between variables.

## Data Availability

All data generated or analysed during this study are included in the published article available on request. More information is available from the corresponding author on reasonable request. Collection of plant material was done in agreement with the PNCal regulation number 02015-046 available at http://www.calanques-parcnational.fr/fr/download/file/fid/1095.
